# Expanding citizen engagement in the secondary use of health data: an opportunity for national health data access bodies to realise the intentions of the European Health Data Space

**DOI:** 10.1186/s13690-023-01182-4

**Published:** 2023-09-12

**Authors:** Marlies Saelaert, Louise Mathieu, Wannes Van Hoof, Brecht Devleesschauwer

**Affiliations:** 1 Department of Epidemiology and Public Health, Sciensano, Rue Juliette Wytsman 14, 1050 Brussels, Belgium; 2 Department of Translational Physiology, Infectiology and Public Health, Ghent University, Merelbeke, Belgium

**Keywords:** European Health Data Space, Health information, Health data, Secondary use, Citizen engagement

## Abstract

The European Health Data Space (EHDS) aims to make the primary use of health data for healthcare provision more continuous, effective, and (cost) efficient. Moreover, it pursues to facilitate the secondary use of health data for purposes such as research, innovation, and policy making. In the context of secondary use, the EHDS legislative proposal (published on 3 May 2022) argues that Member States should develop Health Data Access Bodies (HDABs) whose responsibilities include facilitating the secondary use of health data, issuing data permits, and implementing high levels of accountability and security. In Belgium, the setup in 2023 of a federal Health Data Agency (HDA) that is developing and implementing a policy strategy and framework for the secondary use of health data, aligns well with the responsibilities set out for HDABs. Even though the EHDS aspires the empowerment of citizens, for instance by giving them access to their health data and control over the healthcare professionals who can consult these data, this call for citizen empowerment resonates less loudly regarding secondary use. We think, however, that elaborating and implementing citizen engagement in the domain of secondary use is required to align secondary use with socio-ethical sensitivities, preferences, and values and to provide social legitimacy and ethical solidity to a health data governance system. When implementing the EHDS legislation on a national level, the Belgian HDA and the future HDABs in general might be excellent opportunities to realise this ambition of citizen involvement and empowerment. More specifically, we urge HDABs, firstly, to expand the field of citizen engagement towards the domain of secondary use and, secondly, to respect and facilitate the diversity of citizen engagement. This would offer citizens genuine, continuous and diversified possibilities of involvement and co-creation concerning the development of a solid ethical governance framework for health data.

## Background: making better use of health data

In all domains of society, including health and healthcare, a vast amount of data is produced. These health (related) data do not only sustain primary use, i.e., healthcare provision, they also allow secondary use. This means that these data, combined with data sciences, can serve other purposes such as scientific research or the development of more evidence-based and efficient health policies. However, both at a national and international level, health data are frequently fragmented, and complex (legal, technical, administrative, etc.) structures impede accessing and linking these data. Hence, various barriers hamper secondary use and the full exploitation of health data’s potential.

At the European level, these barriers are addressed by the European Health Data Space (EHDS). Emerging from the European Strategy for Data, the EHDS will be the first domain-specific common European data space. It aims to provide common standards and practices that align with a structural and resilient governance framework for both the primary and secondary use of electronic health data [1–3]. Regarding primary use, the EHDS intends to make healthcare provision more continuous, including cross-border healthcare, as well as more efficient, effective and cost-saving for both patients and the healthcare system. Therefore, accessing and exchanging health data should be improved and main product markets such as electronic health records (EHR) should be harmonised and legally regulated [2]. Regarding the secondary use of health data, the EHDS envisages more robust, data-driven healthcare policies and decision-making and this by virtue of better access to data for researchers, innovators, policy-makers and regulators [2].

We support the initiative but we call for a more coherent consideration of citizen engagement and for expanding and diversifying the concept. More specifically, we argue that Member States’ implementation of the EHDS proposal on a national level may provide the opportunity to truly unfold the idea of citizen empowerment in the context of the secondary use of health data. We will use the Belgian context as an example.

## Secondary use of health data according to the EHDS legislative proposal

The EHDS legislative proposal covers a wide variety of electronic health data collected by both public and private data holders that is considered eligible for secondary use. This includes EHRs, genetic and genomic data, health determinants, health-related administrative data, public health registries, data from research, clinical trials and biobanks, and person-generated data such as data from wellness apps or other wearables [2]. Thereupon, the proposal emphasises a list of common European and legally delineated purposes for which these data can be used by public and private actors [2]. More specifically, the intended purposes of secondary use should comply with reasons of public interest in the area of public or occupational health, public health statistics and policy making, research or innovation activities, educational activities, the development and evaluation of algorithms and artificial intelligence (AI) systems, or the provision of personalized medicine [2]. Anyone who intends to pursue one of these purposes may request and gain lawful access to health data. Hence, the group of data users can include health professionals, researchers, policy makers, and public institutions, as well as commercial companies and the health care industry. Conversely, any kind of secondary use that might result in detrimental measures for natural persons, such as increased insurance premiums, health advertisements, or the development of harmful products, is prohibited [2].

The EHDS proposal also elaborates on governance structures to facilitate secondary use. Member States are requested to develop health data access bodies (HDABs), established as one or more new public sector bodies or relying on existing public sector bodies [2]. This organisation should reflect a Member State’s constitutional, organisational and administrative structure. Hence, the structure will depend on existing public sector bodies or health institutions and whether their tasks and mandates (partially) align with the tasks of the HDABs. HDABs will be responsible for facilitating the secondary use of anonymised or pseudonymised electronic health data (for instance by providing a national data catalogue), issuing data permits to data users, and health data processing for secondary use on the basis of the data permit [2].

## The Belgian health data agency

To realise the secondary use of health data as foreseen in the EHDS proposal, a solid collaboration with and between national stakeholders will be needed, which is reflected in the requirement for Member States to develop HDABs [2]. At present, European countries still differ strongly regarding the development of their health information systems and, hence, regarding their preparedness to join the EHDS [4].

Belgium has a rich health data landscape. However, these data are often fragmented and not sufficiently compliant with the FAIR (Findable, Accessible, Interoperable, Reusable) principles. Requests for secondary use are not harmonised and they require considerable investments, both in terms of time and effort [5]. To counter these obstacles, the legislative proposal concerning the establishment and organisation of a federal Health Data Agency (HDA) has been approved and signed on March 14th 2023. The Belgian HDA pursues the central objectives of facilitating the availability of health (related) data, developing and implementing a policy strategy concerning health (related) data, and stimulating innovation as well as scientific and policy-supporting research [6]. To facilitate the secondary use of health data, the HDA aims, among other things, to function as preferred point of contact regarding secondary use, develop a governance model, provide a data catalogue, facilitate health data access requests, and support the communication between data holders and data users. Other particular tasks include giving advice regarding the standardisation, “FAIRification”, and quality of data, and establishing a *Health Data Academy* providing trainings. Also initiatives such as to create citizen trust concerning the correct use of their health (related) data, are anticipated [6]. The intentions of the Belgian HDA align well with some of the responsibilities set out for HDABs in the EHDS proposal. Especially their facilitating tasks such as providing a national data catalogue, expanding the availability of health data, promoting the development of common data standards and AI in health, and harmonising data request procedures, seem to align well [2, 6].

However, there are also some differences between the ambitions of European HDABs and the Belgian HDA. Firstly, HDABs should take on the role of Trusted Third Party (TTP), as they are the only ones who should hold the encryption keys of pseudonymised data. HDABs will also process electronic health data (including data from other data holders) for various purposes such as collecting, preparing, linking or disclosing these data for secondary use as foreseen in the corresponding data permit [2]. In Belgium, the HDA as it is set up now, will not function as TTP, since other institutions such as the eHealth-platform [7], the Crossroads Bank for Social Security [8] and Statbel [9] are already authorised as TTP and the HDA wants to avoid overlaps with legal competences of other federal bodies [10]. Averting a role as TTP would also align with one of the HDA’s central statements that, even though they want to facilitate health data access for secondary use, they will not hold or process any data themselves, except for metadata and anonymised datasets [10]. Secondly, as foreseen in the legislative proposal, HDABs will be responsible for deciding on data access applications as well as for issuing data permits to access electronic health data for secondary use [2]. Even though the HDA aims to expand its facilitating and data processing role, it now assigns the role of issuing data permits to other federal or regional institution(s) [10].

In the context of both the similarities and differences between the intended tasks of the HDABs and the Belgian HDA, the Belgian Data Protection Authority generally asks for a further clarification of the HDA’s role and its relation to the EHDS proposal [11]. We suggest that in such a clarification, the HDA (and HDABs) would take the opportunity to also clarify and further develop the idea of citizen engagement in the secondary use of health data. This way, they could truly unfold and implement the ambitions that are only tentatively suggested in the EHDS proposal itself, as will be discussed in the next paragraphs.

## Expanding the concept and practice of citizen engagement

Considering the primary use of health data, the EHDS proposal is strongly committed to the empowerment of citizens. Citizens should be able to easily access their data and be thoroughly informed about the use of their health data. Moreover, they should have more control over these data, by deciding whom to share these data with and by adding or adjusting information [2]. It is explicitly stated that safeguarding individuals’ rights over their health data is one of the guiding principles of the proposal [2].

However, there is a remarkable difference regarding the approach of citizens and patients and their potential role in health information governance in the chapter on the primary use of health data versus in the chapter on the secondary use of health data of the original EHDS proposal [2]. The focus on individual control is significantly less prominent in the EHDS considerations on the secondary use of health data. Citizens should be informed about the use of health data and HDABs should aim for a high level of transparency, also towards citizens. Nevertheless, these requirements remain very general [12]. It implies that HDABs should provide information to the public at large on the conditions and legal basis for the secondary use of health data, the applicable rights of citizens regarding secondary use, and the outcomes of projects that include secondary use of health data. HDABs should also host a public website on which a national data catalogue can be consulted, as well as all data requests and permits, available research results, and penalties imposed to data users or data holders who do not comply with the EHDS requirements. However, HDABs should not provide information to citizens regarding the specific projects for which their health data have been used. Only when certain research results could impact persons’ treatment, these persons should be notified by the HDAB [2]. Besides this call to inform citizens regarding the secondary use of health data, the EHDS proposal also urges, yet in a more implicit way, to protect citizens. The rights and freedoms of citizens should be safeguarded and this mainly by implementing a solid governance structure for the secondary use of health data. The backbone of this structure is the delineation of the lawful purposes for secondary use (cf. supra). Moreover, the suggested governance structure includes procedural interventions, such as the use of secure data processing environments, well-defined data permits, and access governance by the HDABs. Also “privacy and protection by design” measures, such as data minimisation, pseudonymisation and anonymisation, are provided [2]. Despite this emphasis on information and protection, the EHDS proposal does not thoroughly consider more active ways of citizen engagement. It repeatedly refers to citizen and patient engagement yet only in a vague and passive way, by stating that HDABs need to engage and cooperate with various stakeholders, including representatives from natural persons and patient organisations. Depending on the topics discussed, patients’ representatives could also be invited to meetings of the EHDS Board, a governing body that should facilitate coordination among Member States concerning both primary and secondary use [2]. However, no further suggestions or concretisations are provided. Hence, when it comes to the secondary use of health data, it seems that citizens and patients are not considered truly relevant stakeholders who might, for instance, in a structural and accessible way indicate their preferences concerning secondary use, help to delineate lawful purposes, or collaborate on a governance framework.

It should be mentioned that the first version of the EHDS legislative proposal, as it has been published in May 2022, has provoked a lot of discussion. This year, a draft report on the EHDS has been published by the European Parliament, as well as a compromise proposal by the Council of the EU. In both documents, issues such as informing data subjects more detailed about the secondary use of their data and the possibility for data subjects to (partially) opt out of the secondary use of health data, are raised [13, 14]. However, it still remains to be seen which amendments will be approved.

### First steps towards citizen engagement in the secondary use of health data

Various initiatives provide initial indications to the Belgian HDA and HDABs in general regarding the development of a more citizen-centric health data governance framework. In 2021, the Belgian King Baudouin Foundation (KBF) surveyed a representative sample of citizens about the secondary use of health data, which showed that many people are willing to share their data when it would benefit society and the common good, an intention termed as data solidarity. Almost three out of four persons would share data for scientific research, whereas almost 50% would share with governmental institutions to support health related policy making. A bit more than half of the people would share data with pharmaceutical companies [15]. These results demonstrate that, although many people might be willing to share their health data for secondary use, this goodwill does not equal a blank cheque and people judge different data users in different ways. A recent citizen e-consultation, Healthy Data, provided more details about these granular preferences as well as indications on how citizens want to be involved in the secondary use of health data. Citizens’ support towards secondary use largely depended on the purpose of the reuse and, accordingly, on the data user. While the majority of the people identified support of the common good as a valuable purpose, merely commercial purposes often raised major concerns. In all cases of secondary use, a balance between the potential benefits and perceived risks (such as re-identification) of secondary use was identified as a key condition. The exact ways of realising this balance were found to depend on the specific purposes of reuse [16]. Moreover, citizens recommended being provided with diverse opportunities for meaningful and active decision-making regarding secondary use. This diversity is essential to accommodate the many different degrees and ways in which citizens want to exercise control over secondary use. Personalised as well as dynamic opportunities of engagement and control support citizens’ preference of directing secondary use into directions considered beneficial and in line with their ethical values [17]. Finally, an initiative such as the We Are project aims to take the leap forward concerning a citizen-centric approach in health data governance. It intends to give citizens more control over their data by owning and actively governing their personal data vault and sharing their data securely with whom they choose [18]. It should be examined if this idea requires an adjusted legal framework to protect citizens’ rights as well as to guarantee the availability and quality of data suitable for research and policy making, yet it clearly hints towards a possible democratisation of the secondary use of health data.

Initiatives such as those mentioned above help paving the way towards citizen involvement and empowerment and can inspire the further development of the Belgian HDA and HDABs in general. In Belgium, efforts have already been made to regulate accessing and sharing of health data for primary use. These strategies combine processes and rules on therapeutic relations, informed consent for sharing health data, and an “access matrix” that defines which groups of health care providers have access to which types of health data [19]. Regarding secondary use, the HDA pinpoints communication and the creation and maintenance of public trust regarding the correct use of citizens’ health data as two of its central tasks. Representatives of patient organisations should also be included in both the Board of Governors and the User Committee of the HDA [6]. Although we support these intentions, we call on the HDA and future HDABs to seize the opportunity they are given by the EHDS proposal and to move one step further. We ask them to further unfold and develop citizen engagement to better understand how exactly the pursued trust can be developed and improved, and on which conditions it depends. Our call towards the HDA and future HDABs is therefore twofold and refers to, firstly, expanding the field of citizen engagement and, secondly, respecting and facilitating the diversity of citizen engagement.

### Expanding citizen engagement towards the secondary use of health data

We strongly encourage future HDABs to expand the focus on citizen engagement that can be identified in the EHDS consideration of primary use to the secondary use of health data and hence to push the boundaries of engagement, active involvement and empowerment into a wider area. Numerous arguments support the active involvement of citizens and the consideration of their perspectives on secondary use. Primarily, extending the idea of citizen involvement from the primary to secondary use of health data aligns better with the fact that primary and secondary use are not always two strictly distinct uses of health data. There might be a strong continuity, especially in the perspective of citizens, for instance when clinical data are used for research as well. The same methods to involve and empower citizens, for instance direct control or specific informed consent, may not always be applicable or feasible in a context of secondary use of health data. However, the same rights, needs and preferences are still at play and should be accounted for in the best possible way. Moreover, patients and citizens are the ultimate source of health data, as well as major funders and end users of innovations that will be realised by secondary use [15]. Hence, information, consultation and participation enable citizens to help mould the development of those products and practices that may impact their lives [20]. Citizen engagement can align secondary use with socio-ethical sensitivities and preferences (for instance regarding purpose, security or confidentiality) and this public acceptability is considered crucial for the social legitimacy and ethical solidity of health data governance systems [21, 22]. It may also increase the realised benefits and innovations, their fair distribution and people’s responsiveness towards them [20, 23]. Finally, citizen engagement is often cited as essentially linked to trust which, eventually, might provide certain actors and institutions “a social license to operate”, i.e. the mandate to decide for themselves on proper, ethical conduct that (as a necessary condition) aligns with societal expectations [15, 20, 23, 24]. Hence, to realise an ethical governance structure for the secondary use of health data, we advise HDABs to involve citizens from the beginning as active partners in the development and implementation of this structure. This would allow them to co-decide whether and to what degree they want to remain involved in this framework or whether they prefer, for instance, a social license system.

### Diversifying the practice of citizen engagement

When offering citizens the option of involvement, we invite the Belgian HDA and future HDABs to thoroughly explore the options of citizen engagement and respect and facilitate its diversity. The Healthy Data e-consultation has shown that there is no uniformity in how citizens want to be involved in the secondary use of health data (cf. supra). Hence, providing a combination of involvement strategies might be the most suitable approach. Examples of current initiatives regarding citizen engagement in health (care) have been mentioned above, yet more inspiration for a diversity of approaches is easily found.

In France, the law on bioethics is revised every seven years and citizens are consulted through various initiatives such as local deliberative events, citizen juries, media campaigns, student and teacher initiatives, etc. The outcomes of these engagement initiatives are discussed in parliament, where politicians have to justify why they follow citizens’ opinions or not and which changes they will implement [25]. In Belgium, an offline citizen forum organised by Sciensano and the KBF resulted in concrete recommendations for the societal use of genomic data, and an organization such as Genomics England includes an Advisory body comprising (relatives of) project participants in its governance structure [26, 27]. Other options include establishing bodies such as data ethics committees or functions such as data ethics officers that, as explicit part of their remit, provide (various) citizen engagement strategies within research or policy-making procedures.

Every engagement strategy has its advantages and disadvantages, yet the fact that some funding bodies make public involvement a requirement to obtain grants suggests that its added value becomes increasingly recognised [28]. One of the key points in offering various engagement strategies and this from the outset of developing a governance framework for secondary use, is that it allows citizens to determine themselves which role they want to play in this framework, instead of nudging them a posteriori towards a socially desired role or contribution. For example, generally promoting ‘data altruism’ while a substantial proportion of the population would not agree to share its data with governmental institutions to support health related policy making (cf. supra, the results of the survey of the KBF [15]) will not realise the pursued aim of societal trust in secondary use. Instead, citizens should be offered various options to collaborate and diverse opportunities to indicate whether, how, and under which conditions they would trust the secondary use of health data and the corresponding framework. This strategy holds the risk of citizens indicating that they do not agree with preconceived notions, established practices, or traditional values regarding secondary use (such as data altruism) or that they do not want to be involved in health data governance or only want to play a minor role. However, truly respecting citizens’ input implies the responsibility to share divergent views as well as the possibility to choose not to be involved in a governance structure or its further implementation and this without suffering any consequences.

## Conclusions

There is no magic formula to involve citizens in the ‘right’ way regarding the secondary use of health data. However, we believe that offering the possibility of involvement in a fundamental, continuous, and diversified way is an indispensable way towards a lasting relationship in which citizens are respected as equal partners. On the brim of implementing the EHDS, we believe that the Belgian HDA and the HDABs should take the opportunity to actually develop these new, diversified ways of constructing an ethical governance framework in which citizens are genuinely and sustainably involved, if and how they want to be.

## Data Availability

Not applicable.

## Abbreviations

AI: Artificial intelligence
EHDS: European Health Data Space
EHR: Electronic Health Records
FAIR: Findable, Accessible, Interoperable, Reusable
HDA: Health Data Agency
HDABs: Health data Access Bodies
KBF: King Baudouin Foundation
TTP: Trusted Third Party
